# Anatomical Features can Affect OCT Measures Used for Clinical Decisions and Clinical Trial Endpoints

**DOI:** 10.1167/tvst.13.4.27

**Published:** 2024-04-19

**Authors:** Donald C. Hood, Sol La Bruna, Mary Durbin, Chris Lee, Yi S. Hsiao, Carlos G. De Moraes, Emmanouil Tsamis

**Affiliations:** 1 Bernard and Shirlee Brown Glaucoma Research Laboratory, Department of Ophthalmology, Edward S. Harkness Eye Institute, Columbia University Irving Medical Center, New York, NY, USA; 2 Department of Psychology, Columbia University, New York, NY, USA; 3 University of Pittsburgh School of Medicine, Pittsburgh, PA, USA; 4 Topcon Healthcare, Oakland, NJ, USA

**Keywords:** optical coherence tomography, glaucoma, reference database, real world

## Abstract

**Purpose:**

To understand the association between anatomical parameters of healthy eyes and optical coherence tomography (OCT) circumpapillary retinal nerve fiber layer (cpRNFL) thickness measurements.

**Methods:**

OCT cpRNFL thickness was obtained from 396 healthy eyes in a commercial reference database (RDB). The temporal quadrant (TQ), superior quadrant (SQ), inferior quadrant (IQ), and global (G) cpRNFL thicknesses were analyzed. The commercial OCT devices code these values based on percentiles (red, <1%; yellow, ≥1% and <5%), after taking age and disc area into consideration. Four anatomical parameters were assessed: fovea-to-disc distance, an estimate of axial length, and the locations of the superior and the inferior peaks of the cpRNFL thickness curve. Pearson correlation values were obtained for the parameters and the thickness measures of each of the four cpRNFL regions, and *t*-tests were performed between the cpRNFL thicknesses coded as abnormal (red or yellow, <5%) versus normal (≥5%).

**Results:**

For each of the four anatomical parameters, the correlation with the thickness of one or more of the TQ, SQ, IQ, and G regions exceeded the correlation with age or disc area. All four parameters were significantly (*P* < 0.001) associated with the abnormal cpRNFL values. The significant parameters were not the same for the different regions; for example, a parameter could be negatively correlated for the TQ but positively correlated with the SQ or IQ.

**Conclusions:**

In addition to age and disc area, which are used for inferences in normative databases, four anatomical parameters are associated with cpRNFL thickness.

**Translational Relevance:**

Taking these additional anatomical parameters into consideration should aid diagnostic accuracy.

## Introduction

Optical coherence tomography (OCT) is widely employed to aid in identifying eyes with glaucomatous damage in the clinic, as well as in clinical studies. In both cases, the most common quantitative measures involve the thickness of the retinal nerve fiber layer (RNFL) around the disc, in the form of either an average (global) circumpapillary (cp) RNFL thickness or the average cpRNFL thickness of sectors of the cpRNFL thickness.^1^^–^^10^

The commercial OCT devices code these regions based on the percentile level of the cpRNFL thickness in a commercial reference database (RDB) of healthy eyes. Red indicates <1% and yellow indicates ≥1% and <5%. The diagnostic accuracy for another set of healthy eyes (for example, from the clinic) will be determined by the cutoff values associated with these yellow and red flags. By definition, the false positives (FPs) based on a cpRNFL metric will include eyes at the lower end of normal cpRNFL thickness. However, diagnostic accuracy can be improved in a particular population by taking into consideration factors contributing to cpRNFL thickness in healthy eyes. For example, to improve the diagnostic accuracy of cpRNFL thickness measures after the OCT scan is obtained, commercial OCT software typically takes into consideration patient age. If age were not accounted for, then more older patients would be flagged as abnormal, although their reduced cpRNFL is due to aging, not pathology.

The purpose of this study was to understand the relationship between anatomical parameters of healthy eyes and measures of cpRNFL thickness at the lower range of healthy. Using the eyes in a commercial RDB, we examined the correlation between four anatomical parameters and OCT measures of cpRNFL thickness and then analyzed the association of these parameters with cpRNFL metrics color-coded as red or yellow by the device software. The results have implications for improving the diagnostic accuracy of the OCT RDB. These implications will be considered in the Discussion section.

## Methods

### Participants

The primary group for analysis included 396 eyes from 396 healthy individuals (57% female) in the Maestro commercial RDB (Topcon Healthcare, Oakland, NJ). Details of the RDB are described in Chaglasian et al.^11^ These eyes were categorized as “healthy” based on a complete ophthalmological exam and visual field tests. As described in Chaglasian et al.,^11^ “The study was registered at the US National Institutes of Health (ClinicalTrials.gov identifier: NCT02447120). Between May and October 2015, 504 participants were enrolled. Institutional Review Board approval was provided by IntegReview IRB (Austin, TX, USA) for the following sites: Illinois College of Optometry, Marshall B. Ketchum University, State University of New York College of Optometry, Western University, and Valley Eye Care Center Medical Associates. Local IRB was used for the University of Alabama School of Optometry and New York Harbor Health Care System sites.”

### Optical Coherence Tomography

**Figure 1.**
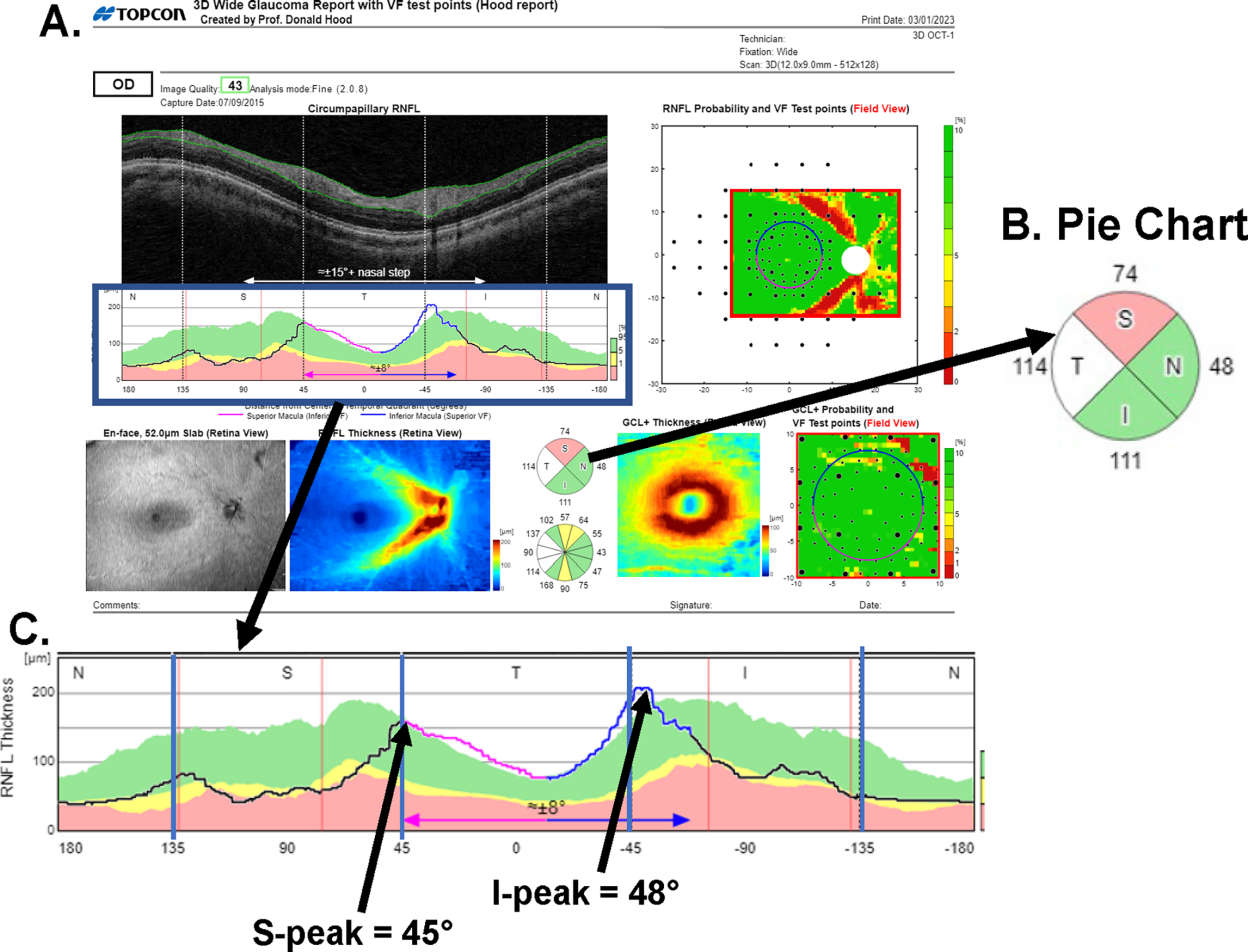
(**A**) The commercial OCT report (Hood Report)^12^^,^^13^ for one of the eyes in the Maestro RDB. (**B**) The numbers indicate the cpRNFL thickness (µm) for each quadrant of the cpRNFL thickness, and the colors indicate the percentile of the thickness after correction for age and disc area: *red*, <1%; *yellow*, ≥1% and <5%; *green*, ≥5% and <99%; and *white*, ≥99%. (**C**) An enlarged version of the cpRNFL thickness map in panel (A) with the location of the superior (S-Peak) and inferior (I-Peak) peaks of the cpRNFL curves indicated by the *arrows*.

All eyes were scanned using the Topcon Maestro instrument and a widefield protocol. Reports were generated with the commercial Maestro software. Figure 1 is the commercial OCT report (Hood Report^12^^,^^13^) for one of the eyes in the Maestro RDB. The numbers in Figure 1B indicate the cpRNFL thickness (µm) for each quadrant of the cpRNFL thickness. The commercial software color codes the quadrants, as well the global (G) cpRNFL thickness, based on the percentile of thickness in the RDB using quantile regression methods that adjust for age and disc area. In particular, red is a cpRNFL thickness that is less than the thickness at the first percentile, and yellow, green, and white correspond to ≥1% and <5%, ≥5% and <99%, and ≥99%, respectively. Here, we focus on three of the four quadrants of the cpRNFL thickness: superior (SQ), temporal (TQ), and inferior (IQ). We omit the nasal, as it is often the site of segmentation errors and accompanying artifacts, and it contains axons from regions well outside the typically tested region of the visual field (i.e., the 24-2 or 10-2 test patterns).^14^

### Anatomical Parameters

We chose four anatomical parameters that we had reason to believe might contribute to a yellow or red flag for one or more of the four cpRNFL thickness metrics (G, TQ, SQ, or IQ):

• *Fovea-to-disc distance (FtoD)*—For FtoD distance, we take the distance (in µm) along the *x*-axis between the center of the fovea and the center of the disc, which is available from the output of the instrument. Based on data from previously published results,^13^^,^^15^ we expected that the thickness of the TQ would be associated with FtoD.
• *Superior peak location (S-Peak)*—The S-Peak is the location (in degrees) along the *x*-axis in Figure 1C of the superior peak of the cpRNFL thickness (Fig. 1C, leftmost black arrow). The superior peak is taken as the largest cpRNFL value between 0° and 180°. Notice that a more positive value indicates a more nasal peak, whereas a smaller positive value indicates a peak that is more temporal.
• *Inferior peak deviation (I-Peak)*—The I-Peak is the location (in degrees) of the inferior peak of the cpRNFL thickness curve (Fig. 1C, rightmost black arrow), which is the largest cpRNFL value between 0° and –180°. We multiple these locations by –1, so that, as is the case for the S-Peak, a more positive value indicates a more nasal peak, whereas a less positive value indicates a peak that is more temporal.

Both S-Peak and I-Peak were obtained automatically from the cpRNFL thickness curve. There is a wide variation in the shape of the cpRNFL thickness curve (Fig. 1C, black curve), and the locations of the peaks of these curves have previously been associated with the locations of the major temporal blood vessel (Fig. 1C, red vertical lines), which have been associated with FPs.^12^^,^^16^^–^^26^

• *Estimated axial length (Est-AL)*—Previous work has documented the influence of axial length on the OCT measures.^27^^–^^38^ Measures of axial length were available for only 276 of the 396 RDB eyes. However, the OCT output includes the position of the mirror in the OCT instrument used to focus the image on the back of the eye for all eyes, and there is a very high correlation (*r*-value of 0.96) between mirror position and axial length. Thus, we used the mirror position as our proxy for an estimate of axial length (Est-AL).

We also included age and disc area, which are currently incorporated into the estimation of the percentile of an individual's cpRNFL thickness before the color of the cpRNFL region is determined. However, we used actual thickness values (see quadrant values in Fig. 1B) for our correlation analyses described below.

### Analysis

Two quantitative analyses were employed. Pearson *r* correlation coefficients (*r*-values) were obtained for each of the six parameters and the four cpRNFL thickness measures (i.e., TQ, SQ, IQ, and G cpRNFL thickness). Independent Student's *t*-tests were employed to test for differences between the eyes with red or yellow sectors versus the eyes with green or white (≥5%). We did not employ a formal test to adjust for multiple tests given the exploratory nature of the study, but we penalized *P* values to less than 0.01 to minimize type 1 errors.

## Results

Table 1 shows the correlation coefficients (*r*-values) between each of the TQ, SQ, IQ, and G cpRNFL thicknesses and the four anatomical parameters (FtoD, S-Peak, I-Peak, and Est-AL). For comparison, the *r*-values for age and disc area (the parameters already accounted for in determining the color of the region) are shown in the first two columns. Notice that all four parameters had *r*-values greater than age and/or disc area for one or more of the four regions (i.e., TQ, SQ, IQ, and G). Thus, all four parameters are possible candidates for correcting the cpRNFL thickness to improve the diagnostic accuracy of cpRNFL measures and/or help clinicians identify FPs. (Supplementary Table S1 has the correlation matrix for the six parameters, and Supplementary Table S2 provides the mean, SD, maximum, and minimum values for the parameters.)

**Table 1.** Correlation *r*-Values (396 Eyes)

Region	Age	Disc Area	FtoD	S-Peak	I-Peak	Est-AL
TQ	−0.24	0.06	**0.36** ^a^	**−0.42** ^a^	**−0.38** ^a^	0.12
SQ	−0.21	0.32	0.08	**0.24** ^b^	0.09	−0.18
IQ	−0.19	0.38	0.09	0.08	**0.35** ^b^	**−0.25** ^b^
G	−0.25	0.42	0.06	0.11	0.21	−0.23

a The parameter had an *r*-value larger than the *r*-value for both age and disc area.

b The parameter had an *r*-value larger than the *r*-value for age or disc area.

To test the hypothesis that a parameter was associated with the cpRNFL color codes, we performed *t*-tests to compare the cpRNFL thickness of each parameter for the eyes with the red or yellow sectors to the cpRNFL thickness for the eyes with green or white quadrants. The results are shown in Table 2. First, age and disc area were not significant for any of the four cpRNFL thickness measures. This was expected, as these parameters are already taken into consideration before the color (percentile) of the cpRNFL region is determined. On the other hand, three of the parameters (FtoD, S-Peak, and I-Peak locations) were highly significant (*P* < 0.001) for one or two of the three quadrant sections. The nature of the relationship between quadrant thickness and the four parameters is considered below separately for the TQ, SQ, IQ, and G thicknesses.

**Table 2.** The *t*-Test Probability Level (Two-Tail) for 396 Eyes

	*P*					
Region	Age	Disc Area	FtoD	S-Peak	I-Peak	Est-AL
TQ	0.870^a^	0.485^a^	**<0.0001** ^b^	**0.0003** ^b^	**0.001** ^b^	0.877^a^
SQ	0.909^a^	0.101^a^	**0.130** ^a^	**0.0007** ^b^	0.180^a^	**0.046** ^c^
IQ	0.733^a^	0.243^a^	0.792^a^	0.144^a^	**0.006** ^b^	**0.029** ^c^
G	0.956^a^	0.849^a^	0.625^a^	0.395^a^	0.082^a^	0.157^a^

a *P* > 0.05.

b *P* ≤ 0.01.

c *P* > 0.01 and < 0.05.

### Temporal Quadrant

**Figure 2.**
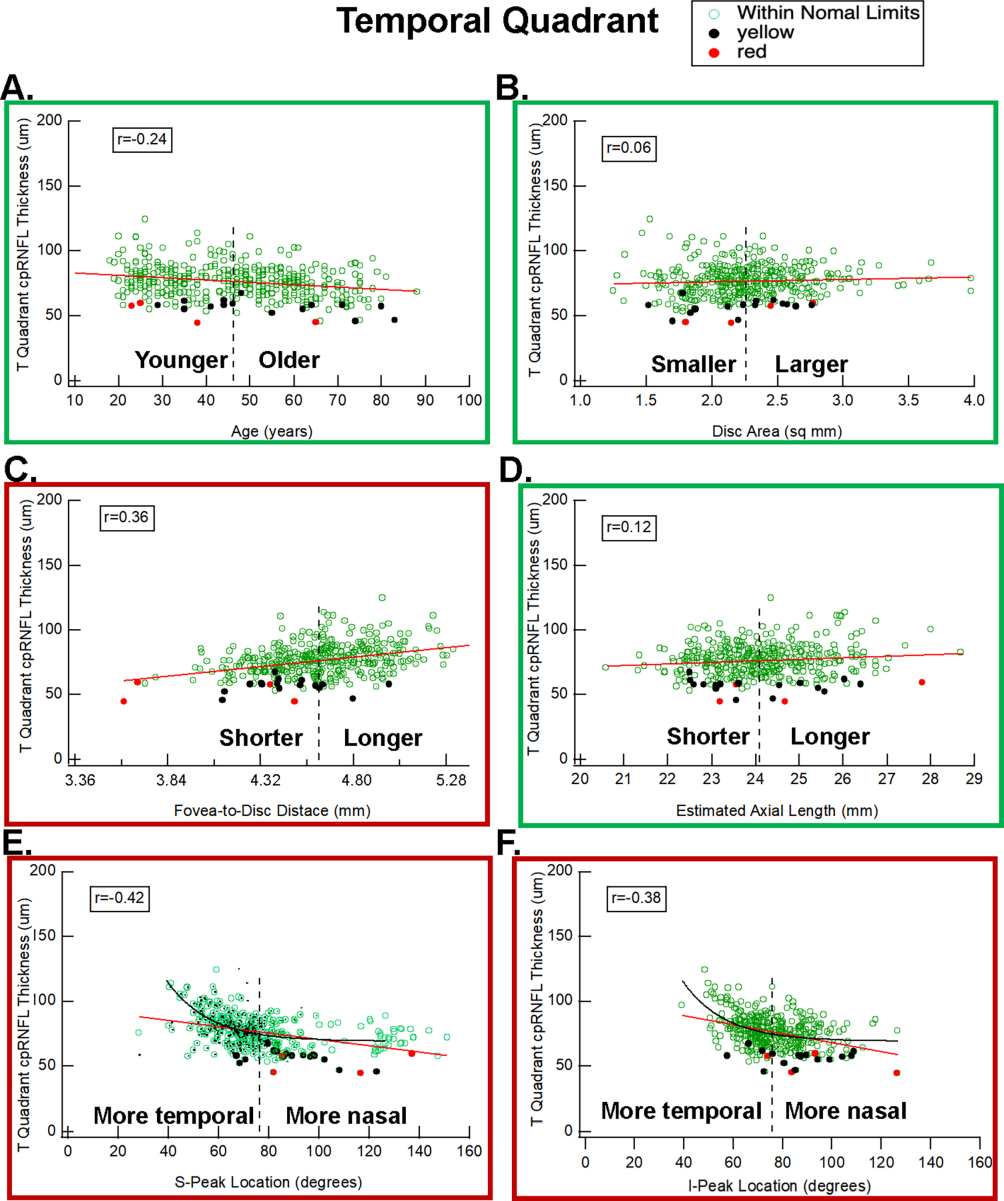
Scatterplots for the TQ thickness versus age (**A**), disc area (**B**), fovea-to-disc distance (**C**), estimate of the axial length (**D**), location of the superior peak (**E**), and location of the inferior peak (**F**) of the cpRNFL curve. The symbols indicate eyes in the <1% (*red*), ≥1% and <5% (*black*), and ≥5% (*green*) percentiles, and the *red lines* are the best-fitting linear regression line. The *black curve* is the best-fitting exponential function.

The *t*-tests indicated that FtoD, S-Peak location, and I-Peak location differed among the 20 eyes with a red (four eyes) or yellow (16 eyes) TQ cpRNFL thickness as compared to the 376 eyes with a white or green TQ (*P* < 0.001). Figure 2 has scatterplots of TQ cpRNFL thickness versus the four parameters, as well as age and disc area. In all panels, the color of a symbol indicates if the TQ sector was red (filled red), yellow (filled black), or green/white (open green). In the case of age (Fig. 2A) and disc area (Fig. 2B), approximately half of the 20 eyes with red or yellow fell on either side of the average values for these factors (vertical dashed line). This is not surprising, as these factors were already taken into consideration when the percentile for the cpRNFL region was determined. In all panels of Figure 2, the red line is the best-fitting straight line, and the red border around the panel indicates that the parameter involved was significant in Table 2.

*A shorter FtoD is associated with a thinner TQ*. The TQ thickness is plotted against FtoD distance in Figure 2C. The correlation with TQ thickness was 0.36 for FtoD, which is greater than the correlations for age (−0.24) or disc area (0.06), the factors currently taken into consideration. In addition, the FtoD of all four eyes with a red TQ sector (Fig. 2C, red symbols) and 12 of the 16 eyes with yellow sectors (Fig. 2C, black symbols) was shorter than the average FtoD; that is, the FtoD values fell to the left of the vertical black dashed line in Figure 2C.

*Compared to the average location of the S-Peak and/or I-Peak, a more nasal location in the peaks is associated with a thinner TQ.* The *r*-values between TQ thickness and S-Peak (−0.42) and I-Peak (−0.38) are greater than the *r*-values for age (−0.24) or disc area (0.06). In addition, the scatterplots in Figures 2E and 2F indicate that the more nasal locations of the peaks are associated with a thinner TQ. As with the FtoD, the scatterplots for S-Peak and I-Peak (Figs. 2E, 2F) suggest that these parameters are associated with TQ thickness. However, the function relating TQ thickness to peak location is clearly not linear. The data are better fitted with an exponential (black curve), which approaches an asymptote value at locations beyond the average location (dashed vertical line).

### Superior Quadrant

**Figure 3.**
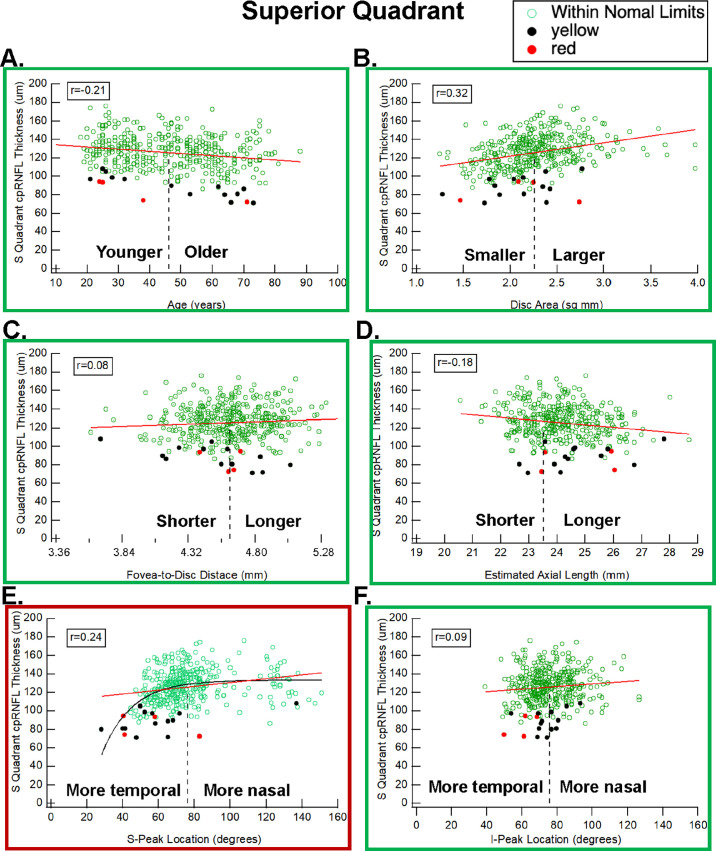
Same as Figure 2 for the superior quadrant (SQ).

The *t*-tests suggest that the S-Peak location, but not the I-Peak location, is a significant factor (*P* = 0.004) differentiating between the 20 eyes with a red (four eyes) or yellow (16 eyes) SQ region compared to the 376 eyes with a white or green. Figure 3 has scatterplots of SQ cpRNFL thickness versus the six parameters.

*A more temporal location of the S-Peak is associated with a thinner SQ.* The *r*-value between SQ thickness and S-Peak (0.24) was greater than the *r*-value for age (−0.21). Notice in Figure 3E that a thinner SQ is associated with a S-Peak location more temporal than average. This is the opposite direction from the relationship between S-Peak location and TQ. Although the scatterplot in Figure 3E suggests that the S-Peak location is associated with SQ thickness, the relationship between SQ thickness and S-Peak location is not linear (red line). It is better fitted with an exponential curve (black), which approaches an asymptote value at locations more nasal than the average location (dashed vertical line).

### Inferior Quadrant

**Figure 4.**
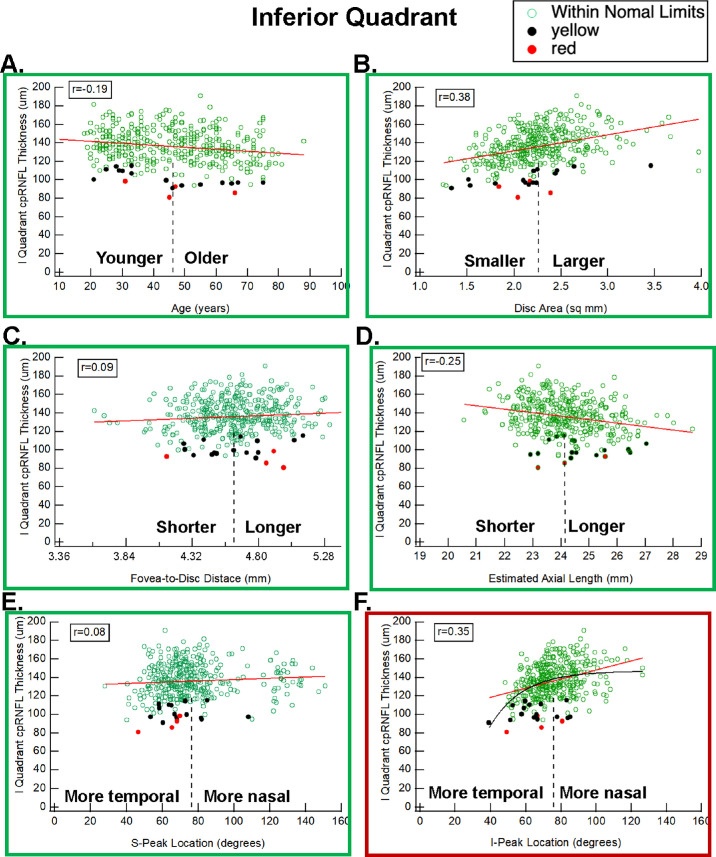
Same as Figure 2 for the inferior quadrant (IQ).

The *P* values for the *t*-test indicate that the I-Peak location (*P* = 0.0006), but not S-Peak location (*P* = 0.144), was highly significant for identifying eyes flagged as red or yellow. Figure 4 has scatterplots of IQ cpRNFL thickness versus the six parameters.

*A more temporal location of the I-Peak is associated with a thinner IQ.* The *r*-value between IQ thickness and I-Peak (0.35) was greater than for age (−0.19) and only slightly less than for disc area (0.38) (Table 1). Notice in Figure 4F that a more temporal location of the I-Peak is associated with a thinner IQ. As is the case with the SQ and S-Peak, this is the opposite direction from the correlation of I-Peak with the TQ. Although the scatterplot in Figure 4F suggests that the I-Peak location is associated with IQ thickness, the relationship between IQ thickness and I-Peak location is not linear (red line) and is better fitted with an exponential (black curve), which approaches an asymptote value at locations beyond the average (dashed vertical line).

### Global cpRNFL

**Figure 5.**
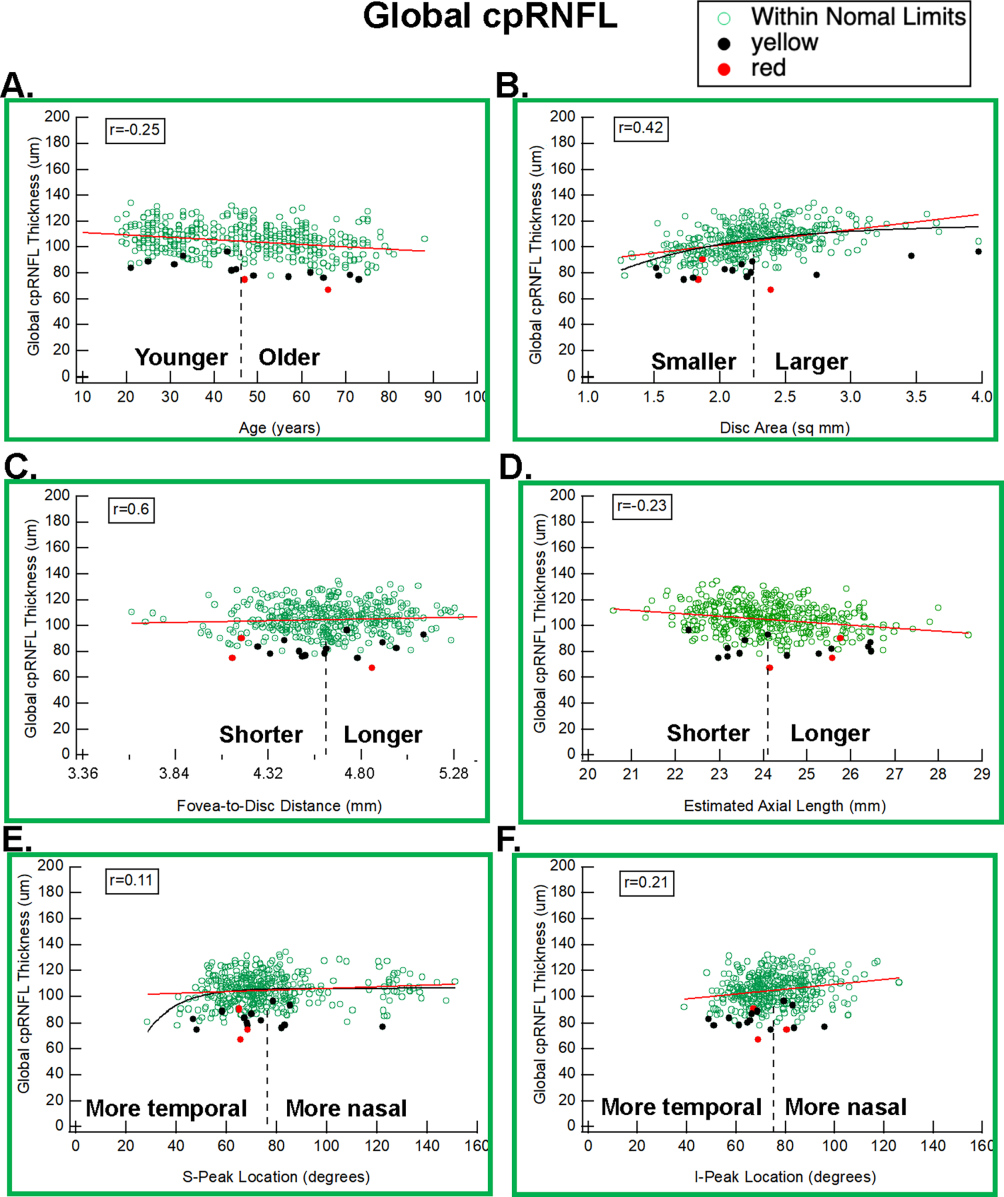
Same as Figure 2 for the global (G) cpRNFL.

None of the *r*-values between the four parameters and G cpRNFL thickness exceeded the *r*-value between G cpRNFL thickness and either age or disc area (Table 1), although the *r*-values for I-Peak and Est-AL (0.21 and −0.23, respectively) were close to that for age (−0.25). Thus, focusing largely on the G cpRNFL thickness can underestimate the impact of these parameters on the quadrant cpRNFL thickness. Further, based on the *t*-test, none of the parameters was significant in differentiating the red or yellow G cpRNFL values from the green or white; the I-Peak had the lowest *P* value (0.082). Figure 5 shows scatterplots of G cpRNFL thickness versus the six parameters.

## Discussion

The purpose of this study was to understand the relationship between anatomical parameters of healthy eyes and OCT measures of cpRNFL thickness and to learn whether these parameters are associated with cpRNFL metrics coded abnormal (red or yellow) in the commercial RDB. The four anatomical parameters studied were fovea-to-disc distance (FtoD), the location of the superior peak of the cpRNFL thickness curve (S-Peak), the location of the inferior peak of the cpRNFL thickness curve (I-Peak), and an estimate of axial length based on the mirror position in the OCT instrument (Est-AL). For each of these parameters, we examined separately the thickness of the TQ, SQ, and IQ of the cpRNFL, as well as the global/average cpRNFL thickness. Commercial software typically categorizes these sectors as white/green, yellow, and red, where the color of the sector depends on the percentile of the cpRNFL thickness of an RDB. In calculating these percentiles, the patient's age and optic disc area are taken into consideration, as they are known to contribute to cpRNFL thickness metrics. To assess the association of a parameter to metrics flagged as red or yellow, we employed *t*-tests to determine if the measure of the parameter was significantly different for eyes with white/green sectors as compared to those with yellow or red sectors. In addition, correlations between the measures of cpRNFL thickness and the four parameters were examined.

There are four findings worth highlighting. First, all four anatomical parameters showed a better correlation (Pearson correlation *r*-values) with one or more of the three quadrants of the cpRNFL thickness than did age and/or disc area, the two factors currently taken into consideration when estimating percentile levels (Table 1). Second, the direction of the correlation was not necessarily the same for different quadrants. In particular, the locations of the S-Peak and I-Peak were negatively correlated with TQ thickness but positively correlated with the SQ and/or IQ thickness. Third, the significant parameter was not the same for different cpRNFL regions; for example, FtoD was highly significant for TQ thickness but not for the SQ, IQ, or G thickness. The Supplementary Material (“Fovea-to-Disc Distance and TQ Thickness”) provides an intuitive understanding of why FtoD is associated with TQ cpRNFL thickness.

### Implications for Improving a Real-World RDB

It should be possible to improve the diagnostic accuracy of OCT cpRNFL measures by taking the four anatomical parameters into consideration. Currently, only age and disc area are included in estimating percentiles of the cpRNFL quadrant and global cpRNFL thickness of the Maestro RDB. The FtoD, S-Peak location, I-Peak location, and perhaps mirror position as a proxy for axial length should be considered as additional factors. We predict that including these parameters will improve diagnostic accuracy in real-world clinical settings. Of course, this predicted improvement in accuracy requires testing with a RDB sampled from a real-world database.

### Implications for Improving Clinical Decisions

It should also be possible to improve the use of the OCT in the glaucoma clinic by taking these parameters into consideration. For example, the percentiles for these four parameters could be added to the report in Figure 1 to alert the clinicians that one or more of the cpRNFL metrics may be a FP. In addition, the iso-RNFL thickness border as seen as the black contour in Supplementary Figure S1 could be added to both the RNFL thickness and p-map to help identify artifacts masquerading as glaucomatous arcuates as shown in figure 17 of Hood et al.^13^

### Possible Limitations

The *r-*values for the parameters that were significant with *P* < 0.001 on the *t*-tests (see Tables 1 and 2) ranged in absolute values from 0.24 to 0.42. By most standards, these *r*-values are in the low to moderate range. However, these values are greater than those for age and/or disc area, which are currently used for correcting percentile levels, which suggest that they add information to the conventional covariates currently used in most OCT devices.

A second limitation is the use of S-Peak and I-Peak locations when actual patients are involved. Optic neuropathy will affect the location of the S-Peak and I-Peak. Therefore, we probably need a proxy for these metrics. The obvious choice is the location of the blood vessels (BVs), which is highly correlated with S-Peak and I-Peak locations in healthy eyes.^16^^–^^19^^,^^39^^,^^40^ However, what is needed is an automated procedure for identifying the locations of the major BVs in the superior and inferior of the temporal half of the disc.

The fact that this study involved only one type of OCT instrument, the Topcon Maestro, is a third limitation. Given that the nature of pre- and post-scanning processing differs across manufacturers, as does the ethnic mix of the RDB, the impact of the four parameters must be assessed separately.

## Conclusions

We identified four anatomical parameters of healthy eyes that are associated with false positives of measures of OCT cpRNFL thickness. Unlike age and disc area, these factors are currently not taken into consideration when determining the percentile level (color codes) of quadrants or global cpRNFL thickness. Our results suggest that taking these four anatomical parameters into consideration should aid the clinician and might improve the diagnostic accuracy of an OCT reference database.

## Supplementary Material

Supplement 1

Supplement 2

Supplement 3
