# Supplementary material for: Anatomical Features can Affect OCT Measures Used for Clinical Decisions and Clinical Trial Endpoints
Source: Transl Vis Sci Technol. 2024 Apr 19;13(4):27. doi: 10.1167/tvst.13.4.27 (PMC11037497; doi:10.1167/tvst.13.4.27)
Supplement: Supplement 1 [file tvst-13-4-27_s001.pdf]

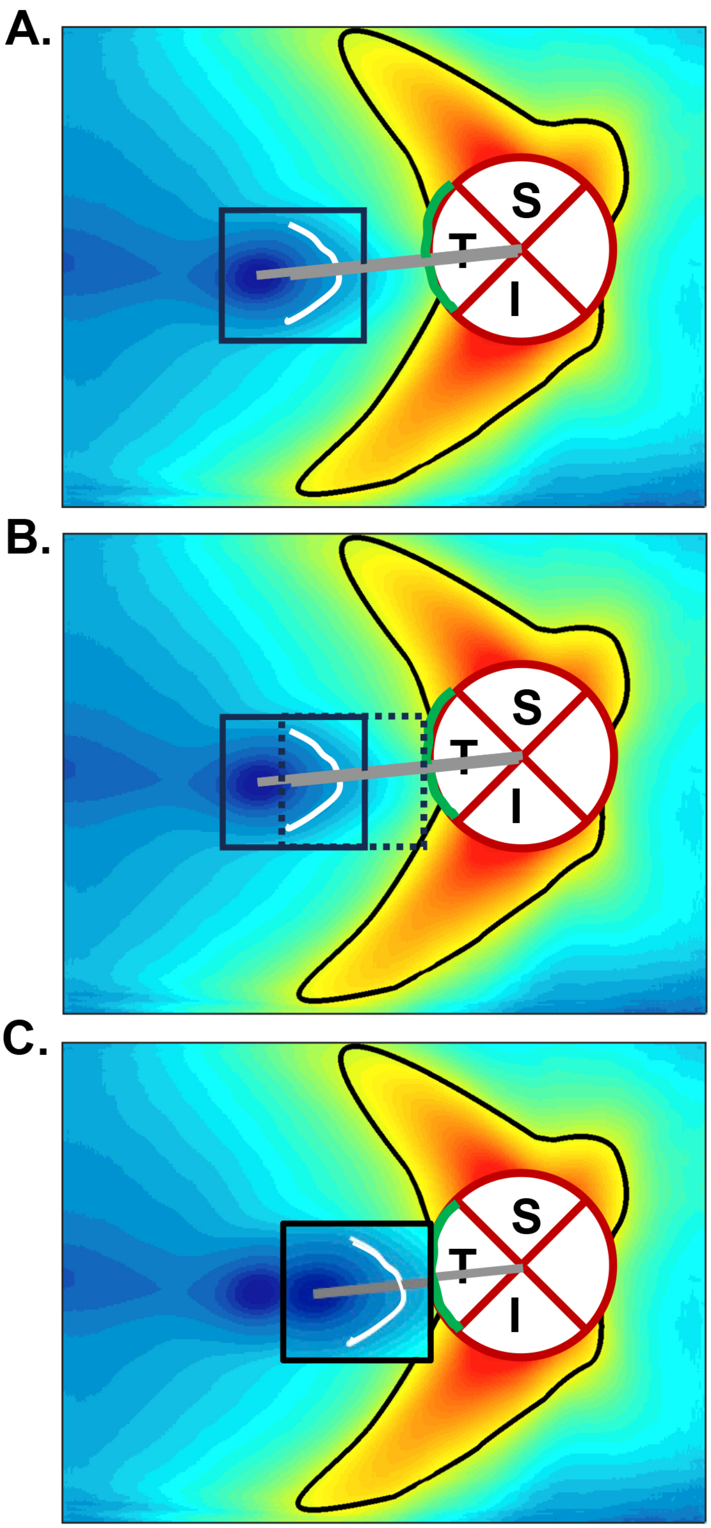

Fovea-to-Disc Distance and Temporal Quadrant (TQ) Thickness:
Based upon the t-tests, FtoD distance was a significant factor for only TQ cpRNFL thickness. To understand the basis, consider the region around the fovea in which the ganglion cell bodies, and their axons, are displayed. Figure S1 shows the average RNFL thickness map of eyes from a different RDB.13 (Note that the color coding of thickness is different from that used in Fig. 1) The gray line extends from the center of the disc to the center of the fovea. The white arc was drawn along an iso-RNFL contour within the thinned RNFL region caused by the displacement of ganglion cells and their axons from the fovea. The exact location of the arc is not important. Suppose, however, we move the fovea closer to the disc and assume that the region within the black rectangle is moved by the same amount, so that it falls in the region of dashed black rectangle in panel B. Now (panel C) the region of thinner RNFL is closer to the disc and overlaps the TQ (green arc) of the circle scan resulting in a thinner TQ. Of course, the schematic is an oversimplification of the anatomical changes due to the development of an eye with a shorter FtoD. However, Fig. S1 illustrates how a shorter FtoD may lead to a thinner TQ.
