# Supplementary material for: Anatomical Features can Affect OCT Measures Used for Clinical Decisions and Clinical Trial Endpoints
Source: Transl Vis Sci Technol. 2024 Apr 19;13(4):27. doi: 10.1167/tvst.13.4.27 (PMC11037497; doi:10.1167/tvst.13.4.27)
Supplement: Supplement 2 [file tvst-13-4-27_s002.pdf]

Table S1. Correlation matrix for the parameters for the 396 RDB						
	Age	Disc Area	FtoD	S-Peak	I-Peak	est-AL
Age	1	-0.08	0.13	0.09	0.11	-0.14
Disc Area		1	0.15	0.12	0.18	-0.17
FtoD			1	-0.19	-0.06	-0.27
S-Peak				1	0.28	-0.1
I-Peak					1	-0.23
est-AL						1
