# Supplementary material for: Anatomical Features can Affect OCT Measures Used for Clinical Decisions and Clinical Trial Endpoints
Source: Transl Vis Sci Technol. 2024 Apr 19;13(4):27. doi: 10.1167/tvst.13.4.27 (PMC11037497; doi:10.1167/tvst.13.4.27)
Supplement: Supplement 3 [file tvst-13-4-27_s003.pdf]

Table S2. Mean, Standard Deviation and Range. (396 eyes)						
	Age (years)	Disc Area (mm)	FtD (mm)	S-Peak (degrees)	I-Peak (degrees)	Est-AL (mm)
mean	46.1	2.26	0.385	76.1	75.7	24.1
SD	16.5	0.41	0.024	21.1	13.4	1.2
max	37	2.34	0.393	74.3	66.0	23.2
min	25	2.19	0.386	60.5	61.1	22.7
